# Defective Osteogenic Differentiation in the Development of Osteosarcoma

**DOI:** 10.1155/2011/325238

**Published:** 2011-02-22

**Authors:** Eric R. Wagner, Gaurav Luther, Gaohui Zhu, Qing Luo, Qiong Shi, Stephanie H. Kim, Jian-Li Gao, Enyi Huang, Yanhong Gao, Ke Yang, Linyuan Wang, Chad Teven, Xiaoji Luo, Xing Liu, Mi Li, Ning Hu, Yuxi Su, Yang Bi, Bai-Cheng He, Ni Tang, Jinyong Luo, Liang Chen, Guowei Zuo, Richard Rames, Rex C. Haydon, Hue H. Luu, Tong-Chuan He

**Affiliations:** ^1^Molecular Oncology Laboratory, Department of Surgery, The University of Chicago Medical Center, 5841 South Maryland Avenue, MC3079, Chicago, IL 60637, USA; ^2^Stem Cell Biology and Therapy Laboratory, The Children's Hospital of Chongqing Medical University, Chongqing 400014, China; ^3^Key Laboratory of Diagnostic Medicine, Chinese Ministry of Education and Affiliated Hospitals, Chongqing Medical University, Chongqing 400016, China; ^4^School of Bioengineering, Chongqing University, Chongqing 400044, China; ^5^Department of Geriatrics, Xinhua Hospital, Shanghai Jiaotong University, Shanghai 200092, China; ^6^Department of Cell Biology, Third Military Medical University, Chongqing 400030, China

## Abstract

Osteosarcoma (OS) is associated with poor prognosis due to its high incidence of metastasis and chemoresistance. It often arises in areas of rapid bone growth in long bones during the adolescent growth spurt. Although certain genetic conditions and alterations increase the risk of developing OS, the molecular pathogenesis is poorly understood. Recently, defects in differentiation have been linked to cancers, as they are associated with high cell proliferation. Treatments overcoming these defects enable terminal differentiation and subsequent tumor inhibition. OS development may be associated with defects in osteogenic differentiation. While early regulators of osteogenesis are unable to bypass these defects, late osteogenic regulators, including Runx2 and Osterix, are able to overcome some of the defects and inhibit tumor propagation through promoting osteogenic differentiation. Further understanding of the relationship between defects in osteogenic differentiation and tumor development holds tremendous potential in treating OS.

## 1. Introduction

Osteosarcoma is the most common primary malignant bone tumor. Most patients with osteosarcoma complain of symptoms for several months and initially present with a pathologic fracture [1, 2]. Although OS can occur in any bone, it frequently involves the metaphysis of long bones where high bone turnover occurs during longitudinal growth spurts [2]. Radiographic imaging, combined with biopsy, is required for definitive diagnosis [2]. However, a problem lies in the detection of the pulmonary metastases, as only around 15%–20% of patients will have radiographically detectable pulmonary metastases, while approximately 80% of the patients will either develop or already have radiographically undetectable micrometastases [1–4]. These pulmonary lesions are responsible for the high mortality associated with OS [1, 2]. Treatment of OS includes surgical resection of both primary and pulmonary lesions combined with radiotherapy [2]. However, due to the high suspicion for micrometastases, nearly all patients will also receive preoperative and postoperative chemotherapy with agents such as cisplatin, doxorubicin, methotrexate, and isofosfamide [1, 2, 5–7]. These agents expose patients to longterm toxicities, including hearing loss, cardiomyopathy, sterility, and hypomagnesemia [2, 8–13]. Even with this aggressive management, OS patients still have a poor prognosis. Patients who present without detectable metastases have a 70% longterm disease-free survival; once a metastasis has been detected, the disease is likely to relapse [1, 2, 5–7]. Thus, there is a critical need to identify metastatic markers that can accurately predict the presence or absence of metastatic disease at the time of diagnosis and provide both prognostic value and potential targets for novel therapies in the future.

Although the etiology underlying OS is poorly understood, the tumors often develop in settings of high bone turnover, such as the adolescent growth spurt [2]. Furthermore, numerous genetic and cytogenetic abnormalities have been associated with OS, including mutations of tumor suppressors and oncogenes, as well as chromosomal amplifications, deletions, rearrangements, and translocations [1, 2, 14]. The most common alterations are associated with chromosomes 1, 9, 10, 13, and 17, or involve the p53 and Rb genes [1]. Given the numerous alterations associated with OS, it is no surprise that no singular consensus mechanism can account for OS tumorigenesis. Recent investigations have focused on the role of osteogenic differentiation in the pathogenesis of OS. This is supported by the similarities between OS tumors cells and primitive osteoblasts [15]. It is plausible that the genetic and epigenetic alterations associated with OS alter the signaling pathways associated with osteogenic differentiation, arresting the cells as undifferentiated precursors. By approaching OS as a disease caused by differentiation defects, we not only acquire a unique understanding of OS pathogenesis, but suggest avenues for developing novel therapies that can target OS differentiation.

## 2. Molecular Biology of Osteosarcoma

### 2.1. Loss of Tumor Suppressors

Both sporadic and inherited mutations to pathways associated with p53 and Rb tumor suppressor genes are associated with osteosarcoma. Rb is a key regulator in the G1/S transition. In its hypophosphorylated state, Rb acts as a tumor suppressor by binding to and inactivating E2F, resulting in cell cycle arrest [16]. Cyclin D1 and CDK4 phosphorylate and inactivate Rb during the G1/S transition, thereby allowing cell cycle progression to occur [16]. Approximately 70% of sporadic OS cases have shown genetic alterations in the Rb1 locus, and individuals heterozygous for a germline inactivation of Rb1 have a 1,000-times greater probability of OS [1, 17–20]. Moreover, inactivation of the Rb1 locus has been implicated as a poor prognostic factor in patients with OS [1, 2, 14]. 

OS development has also been associated with another tumor suppressor in the Rb signaling pathway, p16^INK4A^ [21]. It functions through inactivation of CDK4, causing cell cycle arrest at the G1/S transition. Alterations in p16^INK4A^ cause an inability to regulate CDK4 and the G1/S transition, leading to an uninhibited cell cycle progression that mimics the Rb mutation phenotype. The downregulation of p16^INK4A^ also serves as a poor prognostic factor in pediatric patients with OS [14, 22].

The tumor suppressor gene p53 maps to 17p13, a region that is frequently abnormal in patients with OS [14, 23]. The p53 gene product acts as a transcription factor that regulates cell cycle progression through apoptotic and DNA repair mechanisms, and has been implicated in the pathogenesis of a variety of human cancers, including OS [24–27]. In OS patients, studies have frequently found point mutations, gene rearrangements, and allelic loss at the p53 locus [1]. Furthermore, patients with the Li-Fraumeni syndrome, a disorder characterized by a germline mutation at the p53 locus, have a significantly higher risk of developing OS [28–30].

### 2.2. Induction of Oncogenes

Activation of a variety of oncogenes has been implicated in OS tumorigenesis. The c-Myc oncogene encodes for a transcription factor that regulates both cell proliferation and growth [31, 32]. It is reported that up to 12% of OS tumors have amplification at the c-Myc locus while the expression of Myc appears to be correlated with a higher risk for relapse [1, 33–36]. Furthermore, overexpression of c-Myc in Ink4a/Arf^−/−^ bone marrow stromal cells leads to a malignant transformation [37]. Another oncogene associated with OS is MDM2, an important negative regulator of p53. It encodes a protein that inactivates the N-terminal transactivation domain of p53 and marks it for degradation via polyubiquitination [1, 23–25, 27]. Located at the 12q13 locus, MDM2 has been found to be amplified in up to 10% of OS tumors [38–40]. Finally, CDK4, an oncogene associated with the regulation of cell cycle progression, has shown high levels of expression in up to 65% of low-grade OS [41]. CDK4 forms a complex with cyclin D1 and phosphorylates RB, thereby releasing the E2F transcription factor and promoting cell cycle progression [1]. Other important oncogenes that have been reported in association with OS include, but are not limited to, FOS, ERBB2 and CCND1 [1].

### 2.3. Syndromes Associated with OS

A variety of syndromes show a predisposition to the development of OS. In patients affected by Paget's disease of the bone, approximately 1% will develop OS [42]. Paget's disease of bone results when there is a disconnection between osteoclast and osteoblast activity, resulting in largely deformed bone. Furthermore, Paget's disease accounts for a substantial fraction of patients over 60 years old with OS [42]. Another syndrome that increases the risk of OS is Rothmund-Thomson syndrome, an autosomal recessive disorder that results from a mutation in an RECQ helicase, resulting in photosensitivity, cataracts, and skeletal dysplasias [43]. In one study, 32% of patients with Rothmund-Thomson developed OS, with a tendency to occur at a younger age [43]. Finally, patients with neurofibromatosis 2 (NF2) have decreased expression levels of merlin, an ERM-related protein that acts as a tumor suppressor [44, 45]. Merlin increases the stability of p53 by inhibiting MDM2-mediated degradation, and the loss of merlin in NF2 is thought to destabilize p53 [46]. NF2 heterozygous mice showed a propensity of highly metastatic tumors, including poorly differentiated OS [46].

### 2.4. Dysregulation of Signaling Pathways

Recently, many investigations have focused on aberrations in cell signaling pathways that have been linked to the development of many different human tumors, including OS. One example is the TGF*β* signaling pathway, which involves three distinct proteins (TGF*β* 1–3) that are involved in cellular differentiation, cell growth, and apoptosis [47–50]. In OS tumors, there is significantly higher expression of TGF*β*1 and TGF*β*3 compared to TGF*β*2 [51]. Expression levels of TGF*β*3 strongly correlate with OS tumor progression [51]. Alterations in other signaling pathways that are implicated, but whose roles are less delineated in OS, include Shh, FGFR2, MET/HGF, and BMPs [1, 52–54]. Later, we discuss the signaling pathways associated with the Wnt proteins and Runx2, and their relationship with defects in osteogenic differentiation and subsequent OS tumor development.

### 2.5. Mesenchymal Stem Cell Differentiation

**Figure 1:**
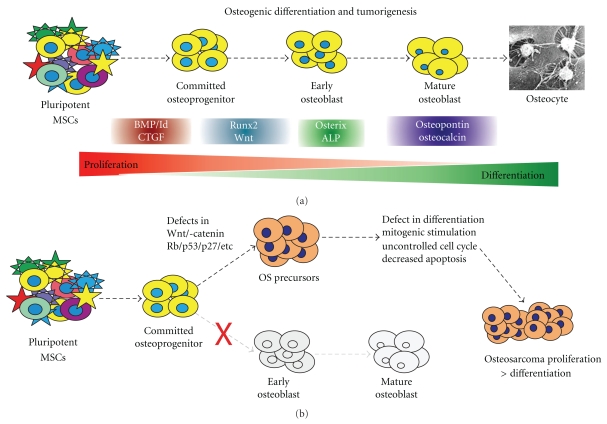
(a) Mesenchymal stem cells (MSCs) progress down the osteogenic differentiation cascade. MSCs are pluripotent bone marrow stromal cells that are able to differentiate into bone, muscle, tendon, and adipose tissue. Osteogenic differentiation of MSCs is a tightly regulated process by different signaling. Bone morphogenetic proteins (BMPs) and their downstream mediators, such as inhibitor of DNA binding (Id) proteins and connective tissue growth factor (CTGF), are early markers in the osteogenic differentiation cascade. Runx2 and Wnt proteins are important regulators of osteoblastic differentiation. Alkaline phosphatase and Osterix are early/middle markers, while osteocalcin and osteopontin are late markers of bone formation. (b) Defects in osteogenic differentiation lead to osteosarcoma (OS) development. If alterations in the MSC differentiation cascade block the progression to terminally differentiated osteoblasts or osteocytes, it is likely that tumorigenic precursors are formed. Such undifferentiated OS precursors would maintain the ability to proliferate and increase the risk for OS development. Although not well understood, some of the potential defects may include genetic and/or epigenetic changes in Wnt signaling, Rb, p53, and p27. These defects may lead to uncontrolled cell proliferation and disrupted differentiation. Thus, these alterations disrupt the delicate balance between proliferation and differentiation, leading to a tumorigenic phenotype.

Mesenchymal stem cells (MSCs) are bone marrow stromal cells that can differentiate into osteogenic, chondrogenic, adipogenic, neurogenic, or myogenic lineages [55–58]. Osteogenic differentiation is a complex, tightly regulated process that is critical for proper bone formation and is influenced by a variety of endogenous and environmental factors [1, 59]. As MSCs pass through each successive stage of differentiation, they are thought to lose their proliferative capacity. Markers of the osteoblastic differentiation cascade include connective tissue growth factor (CTGF) (early), alkaline phosphatase (ALP), Osterix, Runx2 (early/middle), osteopontin (OPN), osteocalcin (OCN), and collagen 1a1 (Col 1a1) (late) [1, 15, 47, 57, 59–64] (Figure 1). 

Many signaling pathways and associated regulatory genes control the complex MSC differentiation cascade [65]. For example, myogenic differentiaion is controlled by factors such as the MyoD and Mef2 family of transcription factors [58, 66, 67]. Commitment of MSCs to the adipogenic lineage is a two-phase process of cell determination and differentiation that is regulated in part by PPAR*γ*, as well as BMPs 4 and 7 [57–59, 68, 69]. Chondrogenic differentiation is regulated by multiple transcription factors and growth factors, such as Sox9, BMP2, BMP7, and FGF2, many of which represent early regulators of the osteogenic differentiation pathway [57, 58]. The factors controlling these pathways are integral in regulating the osteogenic cascade through interpathway cross-talk and feedback cycles. Some of the most important of these molecules include the BMPs, PPAR*γ*, Runx2, and the Wnts (Figure 1). 

BMPs belong to the TGF*β* superfamily of growth factors, which are considered pivotal regulators of early MSC commitment. The osteogenic BMPs include 2, 4, 6, 7, and 9, with BMP 6 and 9 showing the most potent osteogenic activity both in vitro and in vivo [1, 47, 57–59, 70–74]. BMP 4 and 7 also exhibit adipogenic activity, but commitment to the adipogenic or osteoblastic lineage is mutually exclusive [57, 59, 74–83]. These osteogenic BMPs are able to induce undifferentiated MSCs to express many early osteoblast progenitor markers, such as the connective tissue growth factor (CTGF), inhibitor of DNA binding (Id), alkaline phosphatase (ALP) and runt-related transcription factor 2 (Runx2) [57, 75, 76, 84–87]. 

PPAR*γ* is considered the main regulator of adipogenesis. However, it plays a crucial cross-regulatory role in osteoblastogenesis, as PPAR*γ* expression shifts MSC differentiation from the osteogenic to the adipogenic cascade [59, 88]. For example, PPAR*γ*-deficient mice show a lack of adipogenesis with an increase in osteogenic activity [59, 89]. Furthermore, PPAR*γ* seems to be involved in BMP-induced osteogenesis, as PPAR*γ* knockout mice fail to differentiate in response to BMP stimulation [59, 74, 85]. These results suggest that in addition adipogenesis, PPAR*γ* may act as a differentiation regulator in conjunction with the osteogenic BMPs to promote MSC differentiation along an osteogenic lineage.

Runx2 is considered one of the master regulators in MSC osteoblast differentiation [58, 90–92]. Runx2 knockout is fatal in mice, leading to a cartilaginous skeleton without any ossification and delayed chondrocyte maturation [93, 94]. Moreover, Runx2 interacts with numerous transcriptional activators and repressors, which are crucial in osteogenesis, such as Rb, PTH/PTHrP, MAPLK, and histone deacetylases [58, 92, 95–97]. In particular, it is thought to be a critical regulator in the BMP-mediated osteogenic differentiation pathway [98]. 

Wnts are a group of highly conserved, secreted proteins, and are one of the major osteogenic regulators [58, 99–102]. Wnt genes are expressed in developing limbs and the Wnt coreceptor LRP5 has been shown to regulate bone formation [58, 103–105]. Osteoblast maturation is dependent on Wnt proteins, as Wnt deficient cells fail to undergo terminal differentiation in the presence of the hedgehog signaling proteins [106]. Overexpression of a Wnt antagonist leads to the presence of lytic bone lesions, while activation of Wnt/ *β*-Catenin signaling is frequently observed in osteosarcoma [107, 108]. It appears Wnt molecules control both osteoblastic differentiation and cell proliferation while shunting away from chondrogenic differentiation [109].

The effect of terminal differentiation on stem cells is crucial in understanding oncogenesis. When cells progress down a differentiation cascade, they lose their proliferative capabilities in exchange for a differentiating potential. As a result, they are less responsive to growth factors and increasingly susceptible to apoptosis and cytotoxic agents such as chemotherapy [59]. Thus, it is conceivable that tumorigenesis may result from disruptions that prevent terminal differentiation, thereby allowing tumor-initiating cells to retain their highly proliferative precursor cell phenotypes.

## 3. Association between Differentiation Defects and Cancer

Stem cells are undifferentiated precursor cells that have a pluripotent ability to give rise to many different types of tissues. They are defined by their capacity for self-renewal, proliferation, and differentiation into mature cells of a particular tissue. Recent studies have linked undifferentiated progenitor cells with tumorigenesis, and their similar ability to self-renew and proliferate [63]. A crucial aspect of stem cell biology is to regulate the balance between proliferation and terminal differentiation. A dysregulation of this balance in favor of proliferation appears to be associated with many different human tumors (Figure 1).

Both normal stem cells and cancer-initiating cells show a unique ability for self-renewal. Pathways that are normally associated with cancer are also crucial to stem cell proliferation, and vice versa. For example, the notch, Sonic hedgehog, and Wnt signaling pathways are associated with the regulation of the hematopoietic stem cell (HSC) pathway, development and oncogenesis [63, 106, 110–114]. Osteoblast maturation is dependent on Wnt proteins, as Wnt-deficient cells fail to undergo terminal differentiation in the presence of the hedgehog signaling proteins [106]. Overexpression of *β*-catenin in the Wnt pathway can expand the pool of transplantable HSCs from cultured HSCs by propagating stem cell division [62, 63]. Gli1, an intracellular mediator of the hedgehog family, regulates limb bud and osteogenic development [113, 114]. This pathway has also been linked to increased proliferation and tumorigenic transformation [114]. Furthermore, this link is demonstrated in the relationship between epidermal progenitor cells and epithelial cancers [115]. Tumorigenesis is thought to be a summation of multiple events over a period of time. If some of these alterations were blocked to arrest the progenitor cells in undifferentiated, highly proliferative state, it may explain the tumor cells' abilities of self-renewal and propagation [63, 116, 117]. 

Recently, the notion of “cancer stem cells” has taken shape, where a small subset of stem cells fail to undergo terminal differentiation and maintain their proliferative capacities, enabling the tumor to continue to self-propagate and regenerate new cells [63, 118]. As reported by Reya et al., both cancer cells and stem cells maintain tremendous proliferative capacity and display similar phenotypic cellular markers [63]. Additionally, both tumors and stem cells consist of a heterogenous population of cells with different proliferative potentials at various stages of differentiation [63]. Thus, the cancer stem cells may be derived from normal undifferentiated progenitor cells, and are thought to drive tumorigenesis.

Multiple therapeutic interventions have targeted the defects in differentiation and are able to promote terminal differentiation of cancer cells and make them more susceptible to apoptosis. Furthermore, these therapies are able to target a specific tissue type, and therefore avoid the systemic toxicities of most chemotherapeutic agents. For example, in breast cancer the estrogen receptor (ER) blocks differentiation in part through induction of cellular proliferation [119]. Tamoxifen targets this receptor, enabling the cells to undergo differentiation and associated apoptosis [120]. PPAR*γ* ligands and retinoids are able to treat liposarcoma through the induction of terminal differentiation [121–125]. In patients with prostate cancer, antiandrogens and retinoids can promote differentiation, and thus decrease tumorigenesis [126, 127]. Finally, clinical trials have suggested that ARA-C can induce complete remission in patients with AML by inducing the differentiation of myeloid leukemia cells [128]. While there are numerous examples of successful differentiation therapy, one particular example is seen in the treatment of Ewing's sarcoma, another primary bone tumor.

## 4. Ewing's Sarcoma: An Example of Differentiation Defects in a Bone Tumor

Ewing's sarcoma is the second most common malignant pediatric bone tumor [129]. A part of the molecular pathogenesis underlying Ewing's sarcoma is the overexpression of EWS/ETS or EWS/FLI-1 fusion oncogenes that prevent MSC differentiation along the adipogenic and osteogenic lineage [130]. The fusion protein carries out its functions by binding Runx2 and regulating the transcription of the hedgehog mediator Gli1 [130–133]. Silencing of this oncogene leads to the recovery of the MSCs differentiation capabilities [134]. Moreover, expression of this EWS/FLI-1 fusion protein in murine primary MSCs leads to the inhibition of MSC differentiation, and subsequent development of a EWS/FLI-1-dependent Ewing's sarcomas [129]. Collectively, these results suggest that inhibition of MSC differentiation may be crucial to the pathogenesis of Ewing's sarcoma, and that restoration of MSC differentiation potential may be an effective therapy in patients with Ewing's sarcoma.

## 5. Osteosarcoma as a Result of Differentiation Defects

OS cells share many similar features to undifferentiated osteoprogenitors, including a high proliferative capacity, resistance to apoptosis, and similar expression of many osteogenic markers, such as CTGF, Runx2, ALP, Osterix, and Osteocalcin [1, 15, 47, 57, 59–64]. Furthermore, the more aggressive OS phenotypes often resemble early progenitors, while less aggressive tumors seem to share more similarities with osteogenic MSCs that have progressed further along the differentiation cascade [55, 59]. 

Analysis of the expression of osteogenic markers in OS cells demonstrates an early osteogenic phenotype. Alkaline phosphatase, a well-documented early marker of osteogenesis, has a much lower expression in OS tumor cells when compared to hFOB1.19 cells, a committed osteoblastic line [64, 135]. Similarly, the late osteogenic markers osteopontin and osteocalcin are highly expressed in mature, differentiated osteoblasts, but are minimally expressed in both primary OS tumors and OS cell lines [47, 57, 136, 137]. CTGF, a multifunctional growth factor that is normally upregulated at the earliest stages of osteogenic differentiation, also shows elevated basal expression in human OS cells [76]. These results suggest that OS cells likely fail to undergo terminal differentiation, and that the degree of dedifferentiation may correlate with a worse prognosis.

By retaining a phenotype similar to undifferentiated osteoprogenitors, OS cells are able to maintain a capacity for uncontrolled proliferation. For example, it is well established that gradual telomere shortening is an effective mechanism of cell senescence when stem cells become terminally differentiated. However, more than 50% of OS cells utilize an alternative lengthening of telomere (ALT) pathway that prevents telomere shortening, allowing the tumor cells to evade senescence and resemble their stem cell progenitors [138]. As a result, OS cells demonstrate similar rates of proliferation, growth factors responsiveness, and capacity for self-renewal to osteoprogenitor stem cells [139]. Furthermore, the stage at which differentiation is interrupted likely correlates with the aggressiveness and metastatic potential of the various OS tumors. 

The Runx2 and Wnt regulators of osteogenic differentiation are two examples of alterations in the differentiation cascade potentially underlying tumorigenesis (Figure 1). Runx2 is a member of the runt family of transcription factors that has been linked to a variety of human cancers such as leukemia and gastric cancer [98, 140, 141]. Runx2 is a master regulator of osteoblastic differentiation that is consistently altered in human OS [98]. Runx2 and its associated protein p27^KIP1^, are important regulators of the G1 cell cycle checkpoint [98]. Runx2 also physically interacts with the hypophosphorylated form of Rb, a known coactivator of Runx2, to create a feed forward loop that promotes terminal cell cycle exit and the formation of a differentiated osteoblastic phenotype [98]. Additionally, Runx2 regulates BMP-induced osteogenesis, synergistically inducing many terminal differentiation markers [98]. Interestingly, Runx2 has a very low expression in OS cell lines. When considering the role of Runx2 in the cell cycle and terminal differentiation regulation associated with BMPs, Rb, and p27^KIP1^, it is natural that any alterations would lead to uncontrolled proliferation and loss of differentiation. Accordingly, high-grade osteosarcomas show decreased expression of p27^KIP1^, while lower-grade tumors have detectable p27^KIP1^ levels. Furthermore, dedifferentiated OS tumors have significantly lower levels of p27^KIP1^ in comparison to well-differentiated OS. Since OS differentiation status bears prognostic significance, disruptions in the Runx2 pathway and loss of differentiation may be an important step in the development of highly aggressive, less differentiated OS tumors.

Wnt signaling pathway has been implicated in a variety of human diseases [62, 142, 143]. The canonical Wnt pathway involves binding of the Wnt glycoprotein to the transmembrane Frizzled receptor and LRP5/6 coreceptors [61, 144–146]. Ligand-receptor binding prevents downstream phosphorylation of *β*-catenin, allowing it to translocate to the nucleus and activate downstream genes that mediate cell proliferation and differentiation [61]. This canonical Wnt pathway plays a crucial role in osteoblast differentiation, as evidenced by the fact that Wnt3a expression leads to cell proliferation and suppression of osteogenic differentiation in adult MSCs [147]. Multiple aberrations in the Wnt signaling pathway have been associated with OS tumorigenesis [108, 148]. For example, elevated levels of *β*-Catenin, an important regulator of the Wnt pathway, are correlated with osteoprogenitor proliferation and OS metastasis [108, 148]. Furthermore, OS tumors overexpressing LRP5, a Wnt coreceptor, are associated with a poorer prognosis and decreased patient survival [149]. Therefore, it is reasonable to believe that deregulation of the Wnt signaling pathway may lead to OS tumorigenesis by preventing terminal osteogenic differentiation and promoting cell proliferation (Figure 1). 

Given these results, it appears that a lack of terminal differentiation may not only be responsible for OS tumorigenesis, but may also predict its malignant potential. By preventing terminal differentiation, tumors can retain their proliferative phenotypes, responsiveness to growth factors, and overall aggressiveness. If osteosarcoma is a consequence of these differentiation defects, we can focus future research on identifying new therapies targeting cellular differentiation thereby avoiding some of the negative consequences associated with conventional chemotherapy.

## 6. Therapeutic Potential by Targeting Differentiation Defects in OS

Recent investigations have focused on the therapeutic potential to overcome differentiation defects associated with osteosarcoma, and therefore prevent tumorigenesis. Examples of such therapies have been detailed in previous studies and include agents such as nuclear receptor agonists, growth factors, and transcription factors [55, 59, 150–155] (Table 1). In addition to inducing terminal differentiation, these therapies can obviate the need for chemotherapy, thereby avoiding some of the toxicities and chemoresistance associated with current OS therapeutic regimens.

One example of potential OS differentiation agents are the nuclear receptor superfamily of proteins, including PPAR*γ*, the retinoids, and estrogens. Various PPAR*γ* agonists have shown the ability to prevent proliferation and induce osteoblastic differentiation in OS tumor cells [15, 153] (Table 1). When OS cells are exposed to these agents, they exhibit an increased susceptibility to apoptosis, decreased proliferative capacity, and an increase in the expression of differentiation markers such as alkaline phosphatase [59]. Similarly, treatment of OS cells with other members of the nuclear receptor superfamily, such as 9 cis-retinoic acid and all-trans retinoic acid, are able to induce differentiation and growth inhibition in human OS cell lines [150]. When these retinoic acid ligands are combined with troglitazone, a potent PPAR*γ* agonist, there is a strong synergistic effect in inducing cellular apoptosis and differentiation [153]. Another nuclear receptor that has potential in OS therapies is the estrogen receptor. In previous studies, estrogen receptor antagonists, such as tamoxifen, Raloxifene, 17-beta estradiol, and SERMS, are able to inhibit proliferation and induce apoptosis in U2OS cell lines through varying mechanisms [156]. These studies also demonstrated that the decreased cell proliferation was associated with an increase in osteoblast differentiation markers [156].

Another nuclear receptor agonist that has the potential to serve as an OS differentiation inducer is 1,25-dihydroxyvitamin D3 (1,25(OH)2D3) (Table 1). 1,25(OH)2D3 can induce OS differentiation through a p21-dependent pathway [152]. The p21 is a downstream effector of p53 that regulates G1 cell cycle arrest [157]. However, since most OS cells contain absent or nonfunctional p53, this pathway is often interrupted [1]. Osteogenic differentiation of OS cells is associated with the expression of p21 [152]. 1,25(OH)2D3 has been shown to induce the expression of p21, and treatment of three different OS cell lines with exogenous 1,25(OH)2D3 induced cellular differentiation (as measured by ALP and OCN) and triggered apoptosis [151]. Taken together, these results suggest that 1,25(OH)2D3 may prevent OS tumorigenesis by inducing differentiation in a p21-dependent manner. 

An interesting possibility for a differentiation agent is parathyroid hormone (PTH) and parathyroid hormone-related peptide (PTHrP), as they are both able to induce osteoblastic differentiation in MG63 OS cells [155] (Table 1). PTH/PTHrP ligands bind to the G protein family of trans-membrane receptors, and the signal is transduced via a MAPK pathway that leads to the eventual phosphorylation of protein kinase A (PKA) and/or protein kinase C (PKC) [158]. Carpio et al. demonstrated that treatment of MG63 cell lines with PTHrP resulted in elevated levels of ALP and type 1 collagen, suggesting that these tumor cells underwent osteoblastic differentiation. Furthermore, transient transfection of the OS cells with inhibitors of this PTHrP pathway resulted in downregulation of both type 1 collagen and ALP, suggesting that the PTHrP-mediated cellular differentiation is likely a result of activation of the MAPK/PKA/PKC pathway [155]. Interestingly, PTH regulates the oncoprotein c-fos, which is a critical modulator of osteogenic differentiation and malignant transformation [159, 160]. Upregulating the expression of this oncoprotein leads to both malignant transformation and more aggressive tumors [159–161].

Interestingly, as potent osteogenic differentiation regulators BMPs are unable to promote OS cell terminal differentiation (Table 1). BMPs play an essential role in the osteogenic differentiation of MSCs, and exposure of MSCs to the most osteogenic BMPs (2, 4, 6, and 9) result in the expression of osteoblast markers such as ALP, OCN, and OPN [47, 57, 58, 84, 136, 137, 162, 163]. When four different OS cell lines were exposed to these osteogenic BMPs, there was an increased expression of early target genes Id1, Id2, and Id3, but no change in ALP, OCN, and OPN levels [64]. Furthermore, BMP exposure not only prevented differentiation, but actually promoted tumor growth and proliferation [64]. These results suggest that these OS cells may contain defects in the differentiation pathway that are regulated by osteogenic BMPs. Therefore, exogenous administration of BMPs fails to bypass the defects, but instead promotes tumor cell proliferation. However, when the cells were treated with adenovirus expressing Runx2 (even in the presence of osteogenic BMPs), the tumor growth was significantly inhibited, and these cells underwent terminal differentiation and apoptosis [64]. Collectively, these results suggest that Runx2 is able to bypass the differentiation defects that are downstream in the cascade from the BMPs, and thus, able to inhibit tumor progression through the induction of osteogenic differentiation (Table 1). 

## 7. Concluding Remarks and Future Directions

Osteosarcoma is a complex disease whose etiology is likely from multiple sources, including rapid bone proliferation, an accumulation of mutations, and possible defects in differentiation. Recent investigations have focused on the factors regulating the osteogenic differentiation cascade of mesenchymal stem cells. Alterations in other differentiation pathways have already been established as critical etiologies in the pathogenesis of other cancers, such as breast, prostate, and the hematologic system. We have had success in overcoming these differentiation defects in these cancers, leading to the inhibition of the tumor cells with uncontrolled proliferation. We have recently shown that osteosarcoma, at least in part, results from defects in the osteogenic differentiation cascade. OS tumor cells share many cellular and morphologic features with undifferentiated osteogenic progenitors. As a result, osteogenic factors such as BMPs, are not able to bypass these defects, leading to cellular proliferation and tumor growth. Late osteogenic regulators, such as Runx2 and the retinoids, are able to overcome these defects and stimulate progression through the differentiation cascade. Further understanding of the relationship between defects in differentiation and tumor development holds tremendous potential in developing novel therapies to treat OS.

## Figures and Tables

**Table 1:** Summary of some currently used differentiation agents in human osteosarcoma cells. These differentiation agents are in general nonspecific differentiation-promoting agents, and are able to promote osteogenic differentiation in mesenchymal stem cells. These agents can inhibit the proliferation and induce apoptosis in OS cells.

Class	Target	Ligand	Possible mechanism	References
	PPARy	Troglitazone	(i) Increased susceptibility to apoptosis	Haydon 2007, Logan 2004 [15, 146]
		Ciglitazone	(ii) Decreased proliferative capacity	Scotlandi 1996 [54]
		Pioglitazone	(iii) Increased differentiation (ALP Activity)	Deng 2008 [58]

Nuclear		9 cis-retinoic acid	(i) Induced morphologic differentiation	Haydon 2002, Logan 2004 [15, 146]
receptor	Retinoids		(ii) Inhibited anchorage-dependent growth	Luu 2004 [143]
ligands		All-trans retinoic acid	(iii) Decreased proliferative capacity	

	Estrogens	Tamoxifen	(i) Increased apoptosis	Hoang 2004 [149]
		Raloxifene	(ii) Decreased cell proliferation	
		17-*β* Estradiol	(iii) Increased osteoblastic differentiation markers	

	Vitamin D	1,25-dihydroxyvitamin D3	(i) Decreased cell proliferation (increased p21 expression causing G1 arrest)	Cadigan 1997 [144]
				Wodarz 1998 [145]
			(ii) Increased differentiation (ALP, OCN)	
			(iii) Increased apoptosis	

	Parathyroid	Parathyroid	Increased differentiation via MAPK	Iwaya 2003 [148]
Hormone (s)	hormone	Hormone-related	pathway (ALP, Type 1 Collagen)	
		peptide (PTHrP)		

		BMP2	(i) −Runx2: increased cell proliferation, no differentiation in OS cells	Reya 2001 [63]
	Bone	BMP4		
Growth	morphogenetic	BMP6	(ii) +Runx2: decreased cell proliferation, increased OS cell differentiation	
factors	proteins	BMP9		
